# Perception and Experience of Independent Consultations in Primary Healthcare among Registered Nurses in Kazakhstan: A Qualitative Study

**DOI:** 10.3390/healthcare12151461

**Published:** 2024-07-23

**Authors:** Zhanar Dostanova, Lyudmila Yermukhanova, Aurelija Blaževičienė, Zaure Baigozhina, Maiya Taushanova, Indira Abdikadirova, Gulnar Sultanova

**Affiliations:** 1 Department of Public Health and Health Care, West Kazakhstan Marat Ospanov Medical University, Aktobe 030019, Kazakhstan; yermukhanova@zkmu.kz (L.Y.); dr.taushanova@zkmu.kz (M.T.); a.indira.t@bk.ru (I.A.); g.sultanova@zkmu.kz (G.S.); 2 Department of Nursing, Lithuanian University of Health Sciences, 44307 Kaunas, Lithuania; aurelija.blazeviciene@lsmu.lt; 3 School of Nursing, Astana Medical University, Astana 010000, Kazakhstan; baigozhina.z@amu.kz

**Keywords:** advanced nursing practice, qualitative study, primary healthcare, patient satisfaction, nurse’s function

## Abstract

(1) Background: The nursing profession has undergone a significant transformation not only in a formal context but also in terms of the increased educational competencies required of nurses and their preparedness to adapt to evolving roles and statuses. The aim of our study was to examine the experience of advanced practice nurses who conduct independent consultations on patients and to identify the main challenges they face in their practice. (2) Methods: We carried out 22 semi-structured interviews with nurses responsible for conducting independent consultations across three urban polyclinics located in Aktobe, Almaty, and Astana. All interviews were audio-recorded, transcribed verbatim, and subjected to qualitative content analysis. (3) Results: The content analysis revealed three main themes: “People awareness of the role of an independent nursing appointment”, “Adaptation of the work environment”, and “Confidence of nurses to provide independent services”. (4) Conclusions: Limited competency among nurses and the absence of quality metrics for autonomous nursing consultations present substantial obstacles to assessing and enhancing the quality of care delivered by nurses in their independent roles. Developing and implementing quality indicators for independent nursing appointment, as well as additional training within the framework of interprofessional continuous education for nurses, are important steps toward enhancing the effectiveness, safety, and accessibility of nursing care.

## 1. Introduction

Issues related to human resources in healthcare are crucial globally for both structuring and providing medical services. Effective functioning of healthcare systems and their ability to address emerging challenges depend significantly on the sufficiency of medical personnel, their competency levels, and the creation of conditions that promote motivation and professional development among healthcare workers [1].

In recent decades, substantial transformations have taken place in the nursing domain, particularly regarding aspects such as salary compensation, the demand for the profession, the responsibilities of nurses, the integration of technology, nursing education methodologies, holistic patient care, and workload intensity. Nurses are becoming increasingly popular and in demand. For many years, members of the nursing community were considered mere physician assistants; but today, nurses themselves are high-level professionals. The nurse–patient relationship often allows us to delve more deeply into the patient’s evolution and adherence to the prescribed treatment, often fostered by close-ness and the time factor of dedication. These factors encourage the nurse to acquire more information about each patient in front of the medical professional [2,3]. Consequently, there has been a growing discourse within the professional arena regarding the augmentation of nursing functions as a strategy to elevate the overall efficacy of healthcare system operations [4].

Existing research confirms that advanced practice nurses (APNs) have played a significant role in improving the quality of healthcare services, particularly in ensuring higher patient satisfaction compared to general practitioners, through longer consultations, comprehensive provision of medical information and recommendations, as well as enhancing treatment effectiveness and reducing healthcare costs. APNs spend more time on patient consultations. This allows them to discuss health issues in greater detail, answer questions, and build trust, which enhances patients’ perception of care quality. These nurses often provide more detailed and comprehensive information about illnesses, treatments, and prevention strategies. This helps patients better understand their conditions and make informed health decisions. Improving the quality of treatment and preventive measures reduces the number of repeat visits, hospitalizations, and the need for expensive procedures. Consequently, this leads to lower overall healthcare costs. Due to their holistic approach and high level of expertise, advanced practice nurses significantly contribute to the improvement of healthcare quality and patient satisfaction [5,6,7,8,9,10,11].

An examination of international practices suggests that adopting patient-centered healthcare models results in heightened workloads for nurses, necessitating an expansion of their skill set. Developed nations employ nurses in diverse capacities, encompassing preliminary consultations, prescription authority, and overseeing patient care coordination. Empowering nurses with additional responsibilities yields several advantages, including shorter appointment wait times, prompt referrals to specialized care, decreased physician workloads, enhanced patient contentment, and a catalyzation of professional growth within the healthcare workforce. Nevertheless, potential drawbacks may entail conflicts of interest between the physician and nursing domains. In numerous advanced nations, within the framework of healthcare system restructurings grounded in patient-centered paradigms, nurses shoulder considerable workloads: their responsibilities and skill sets evolve, and their roles broaden. Patient-centered approaches aim to enhance the caliber of healthcare provisions, requiring the careful allocation of duties between physicians and mid-level medical personnel [10]. As an illustration, in Ireland, revisions to the Irish Medicines Board Act alongside the implementation of updated professional criteria have empowered nurses with prescribing privileges, thus extending their scope of practice. Research outcomes indicate that this initiative has facilitated prompt referrals to specialized care, diminished wait times, lowered hospitalization rates, alleviated physician burdens, heightened public satisfaction with healthcare provisions, and stimulated healthcare workers’ enthusiasm for professional advancement [11].

In Finland, Germany, the United Kingdom, Switzerland, and other nations, nurses already operate autonomously, devoid of physician oversight. This entails conducting preliminary patient evaluations (basic medical assessments), administering treatment within their scope of practice (a restricted selection of medications and therapeutic interventions), monitoring patient statuses (including postoperative phases), offering support to individuals with chronic ailments (like diabetes, asthma, heart failure, mental health disorders, etc.), and managing complex conditions (encompassing oncological and infectious diseases). Additionally, nurses are empowered to make decisions regarding referrals to primary care physicians or specialists, or to other medical institutions or departments, as well as conducting home visits for designated patients [12,13]. In several countries, such as Belgium, Hungary, Germany, Italy, Lithuania, Poland, and Slovenia, nurses have been granted expanded roles through additional training. They are now capable of providing lifestyle advice, promoting health, conducting screening programs, assisting patients in adhering to medical prescriptions, and educating individuals or groups on health-related issues [14].

The Ministry of Healthcare of the Republic of Kazakhstan has identified the reform of nursing as one of its future strategic goals. The main objective has been designated as the need for highly qualified nursing personnel possessing globally recognized competencies [15]. In Kazakhstan, the organization of nursing services is currently inadequate and necessitates substantial enhancements and modifications in nursing service administration approaches. Nurses are solely tasked with executing physician directives and are not involved in autonomous nursing practice. The substandard quality of nursing care results in stressful circumstances and patient discontent, thereby compromising the fundamental tenet of healthcare provision, “patient safety”. As per the directives outlined in Order No. 419, issued by the Minister of Healthcare of the Republic of Kazakhstan on 4 July 2018, a pilot initiative has been initiated to implement a novel model of nursing service within healthcare establishments across Kazakhstan. This project is associated with the expanded functionality of nurses with both practical and academic bachelor’s degrees in nursing, whereby nurses will not only execute physician orders but also act as equal partners with physicians. After successful piloting, this project was approved and implemented in practical healthcare. The introduction of the “advanced practice nurse” model, intended to enhance nursing services, has not been universally adopted across all clinics in Kazakhstan, consequently limiting the availability of autonomous nursing consultations in certain areas.

In Kazakhstan, an APN is a practitioner possessing post-secondary or advanced education in nursing, tasked with executing extended responsibilities within the domain of nursing care. The main qualification requirement for an advanced practice nurse in Kazakhstan is post-secondary education in the specialty of “Nursing” (applied bachelor’s degree) or an academic bachelor’s degree [13]. Academic bachelor’s degree is a higher education, the educational programs of which are aimed at training personnel with the award of the academic degree “bachelor” in the relevant specialty. “Applied Bachelor” is a qualification that is awarded to a graduate who has mastered the basic educational program at the bachelor’s degree level, who has the competence to solve technological problems in various fields of socio-economic activity and is ready to start working immediately after graduation. The essence of the applied bachelor’s degree is to raise the status of vocational education, equating some specialties with higher education that meet innovative requirements. The Applied Bachelor’s degree in Nursing is the training of practice-oriented nursing professionals who are able to critically analyze information, effectively manage resources, and provide safe patient-centered care based on evidence-based nursing practice.

APN professionals, representing a new cadre, will administer autonomous nursing services under the delegated authority of physicians, encompassing individual consultations, ongoing monitoring, advisory services, educational interventions, and home-based care within disease management protocols and a comprehensive, progressive home care framework. Additionally, they will be involved in health promotion initiatives, disease prevention efforts, screenings, vaccinations, and a spectrum of diagnostic and therapeutic procedures [16].

The aim of our study was to examine the activities of advanced practice nurses who conduct independent consultations on patients and to identify the main challenges they face in their practice.

## 2. Materials and Methods

In our study, we employed a qualitative descriptive design based on semi-structured individual interviews. This method explores and provides us with a deeper and more detailed understanding of nurses’ issues from their own perspective in real-life situations [16]. Based on the conducted systematic literature review in medical publication databases, a questionnaire consisting of 18 questions was developed, allowing us to gather all valuable information in terms of quality research criteria (Table 1). We obtained a certificate of entry into the State Register of Rights to Objects Protected by Copyright No. 40387 dated 10 November 2023, “Semi-structured Interview for Nurses Undertaking Autonomous Consultations within Primary Healthcare Settings”. The semi-structured interview encompasses inquiries related to socio-demographic particulars, job scope and characteristics, preparedness for autonomous delivery of diverse medical services, criteria for evaluating autonomous nursing consultations, and queries aimed at uncovering workplace concerns.

### 2.1. Study Setting

This study was conducted at City Polyclinic No. 3 in Aktobe, City Polyclinic No. 5 in Almaty, and City Polyclinic No. 5 in Astana. The selection of these polyclinics was due to the fact that it was these locations where the practice of independent nursing appointment was implemented.

### 2.2. Participants

Participants were purposively selected from the chosen city polyclinics as these healthcare organizations had implemented independent nursing appointment. The target group of the study consisted of advanced practice nurses who conduct independent patient receptions. The sample size was determined by achieving saturation [17]. Thus, the number of participating nurses amounted to n = 22. Inclusion criteria: Nurses with academic and applied bachelor’s degrees who conduct independent appointment. Exclusion criteria: Nurses with technical and vocational education.

### 2.3. Data Collection

Data collection took place from April to August 2023. Interviews with nurses were conducted individually in a quiet room within the respective polyclinic, ensuring no external access. The total duration of interviews ranged from 15 to 40 min. For this study, mobile voice recorders were utilized as the data collection method. Recording the interviews helped preserve all participants’ words and comments, as well as the researcher’s questions. This approach minimized the risk of data loss during analysis, allowing for the collection of comprehensive information from each participant and facilitating qualitative data analysis. Additionally, it enabled the researcher to observe the participants during the interviews, establish eye contact without losing data, and ensure a thorough understanding of the collected information [18].

### 2.4. Data Analysis

All audio recordings were transcribed verbatim into text data using Microsoft Word version 14.0. Content analysis was conducted to analyze the obtained information, employing an inductive approach [19,20]. This methodology was chosen because it focuses on qualitative research in the field of nursing and ensures both reliability and scientific rigor. We followed these steps to create codes through direct and inductive assessment of the data: Researchers listened to the interviews multiple times and transcribed them word for word. Paragraphs, sentences, and words were considered as units of meaning. A unit of meaning is a set of words and sentences linked by content and classified based on their context and content. The texts were reviewed multiple times to identify words containing key concepts or units of meaning and to extract initial codes. Codes were reviewed multiple times in a continuous process, from extraction to labeling. Similar codes were merged, classified, and labeled, and subcategories were defined. Extracted subcategories were ultimately compared and, if possible, merged to form main categories. As a result, subcategories were formed, leading to the emergence of the main theme. To enhance the credibility of the study, the themes and subthemes were reviewed and validated by other researchers.

**The stages of conducting qualitative research:**
Stage 1. Data collection through semi-structured interviews.
Stage 2. Developing a written text based on interview data.
Stage 3. Definition of analysis units, such as paragraphs, sentences, or semantic units.
Stage 4. Data reduction using coding or classification system.
Stage 5. Grouping codes by categories and themes.
Stage 6. Ensuring interpretation of relationships constituting descriptive or explanatory basis.
**Rigor.** To assess rigor in this study, trustworthiness criteria were employed [20]. These criteria encompass dependability, credibility, confirmability, and transferability. To guarantee dependability, the authors meticulously detailed any modifications in data collection and examined how these changes could impact the outcomes. Independent analysis of the data was conducted to maintain credibility. They deliberated on the necessity of additional information. Pre-collection discussions about experiences and perceptions of the research topic helped in identifying inherent biases. To asses confirmability, the interviewer paid close attention to the supervisors’ responses and sought clarifications when needed. To improve transferability, the participants, context, and analysis process were thoroughly described.

## 3. Results

The results of the individual interviews included those obtained from collecting demographic data of the research participants (age, gender, education, tenure in the position of advanced practice nurse). The demographic data show that the majority (n = 20) of participants were females, while the rest (n = 2) were males. This can be explained by the fact that nursing remains a profession predominantly occupied by women. Characteristics of the participants are shown in Table 2.

As a result of the content analysis, three main themes were identified: (1) “People awareness of the role of an independent nursing appointment”, (2) “Adaptation of the work environment”, and (3) “Confidence of nurses to provide independent services”. The main themes are depicted in Table 3.

### 3.1. People Awareness of the Role of an Independent Nursing Appointment

Low patient attendance at nurse-led clinics—was primarily attributedto insufficient knowledge or misunderstanding among patients about who a nurse practitioner is—is associated with the recent introduction of the new nursing model and limited awareness of nurse-led consultations at the primary healthcare level. Because nurses in Kazakhstan were not previously considered as partners to physicians, did not make independent decisions, and were largely perceived as their assistants, some uncertainty still exists among patients, leading them to prefer seeing physicians. The statements of the participants are presented below:

“Since the nurse-led clinics were recently established, patients are still not fully aware of the nurse-led consultations. Therefore, we ourselves inform, explain, and invite them, after which they come for appointments”.

Nurse-2.

“It’s difficult to attract patients for appointments; the number of patients we see per day is very low”.

Nurse-5.

“The population still does not understand what independent consultations mean. Even if we publish this information fully on Instagram, many still do not understand. We gradually explain to patients. They are used to the idea that only doctors should provide care. But everyone is slowly getting used to me. It takes time”.

Nurse-7.

Nurses note that patients’ attitudes towards independent nursing consultations can be both positive and negative. Some patients may welcome this opportunity and trust the experience and expertise of nurses, allowing them to provide medical care:

“Patients are satisfied; sometimes they say, ‘It’s a good idea. When we stand in line for the doctor, we’re always arguing among ourselves, but when we come to you, we get the medication without any problems.’ Then, the doctor has very little time, only 15 min, but with us, we sit for 30 min, talk, receive information about diets, and we have close contact with each other”.

Nurse-9.

Other patients, however, may experience some mistrust or anxiety due to the lack of direct medical supervision:

“In general, our patients do not take us seriously; they regard us as ordinary primary care nurses. I believe they will get used to it over time; they think only doctors should examine them, only doctors can provide consultations”.

Nurse-13.

“Often, young nurses are treated skeptically and not trusted. Well, we have been working for a long time; people treat us differently”.

Nurse-15.

### 3.2. Adaptation of the Work Environment

During their practical work, nurses encountered obstacles such as a large number of medical information programs and the lack of integration between them, which in turn hindered their work:

“Challenges: working with programs, i.e., filling out one questionnaire for one program; for example, there are patients who live with 2-3 diagnoses, meaning that you fill out one form first, then the second form, and it needs to be uploaded. Then, you need to open the Disease Management Program (DMP). Filling all of this out takes a lot of time. And to prescribe medication, there is a separate program. For this, you also need to print, stamp, and this takes a lot of time”.

Nurse-16.

The nurses noted that the lack of separate offices for each nurse conducting independent consultations does not allow them to fully address all patient complaints, thereby reducing the objective assessment of nursing diagnosis:

“Of course, it’s not feasible for every nurse to have their own separate office, but ideally, each nurse would have their own space. For example, we currently share one room with four nurses, and each patient comes to see us there. Some patients want to fully open up; they sometimes come not just for medical issues but to talk about their personal problems at home. They want to open up and share, but with other people nearby, they do not always feel comfortable doing so”.

Nurse-17.

### 3.3. Confidence of Nurses to Provide Independent Services

Nurses’ competence refers to their ability to provide high-quality medical care based on their knowledge, experience, and professional skills. However, ever since this model of advanced practice nursing has been implemented, many nurses have since recognized their lack of sufficient knowledge and skills when independently managing patients:

“We still need a lot of training; for example, to listen to the lungs like a doctor, we do not yet have enough knowledge. But now we are slowly learning to examine the axillary glands and lymph nodes, and I want to learn more about medications”.

Nurse-18.

Understanding and competence in medication management are crucial aspects of nurses’ independent practice. This involves knowledge of various medications, their dosages, side effects, interactions with other drugs, and patient-specific factors such as allergies or health conditions. Insufficient competence in pharmacology can pose serious risks to patients, including potential complications, incorrect prescriptions, or inadequate recommendations. Therefore, ongoing education and knowledge updates in pharmacotherapy should be a priority for nurses to ensure the safety and effectiveness of patient care, as well as to grant nurses the authority to prescribe medications:

“My knowledge is certainly insufficient, especially regarding medications; sometimes I struggle”. “…No, it is not enough. For example, I have a poor understanding of pharmacological drugs and how they work. We do not prescribe medications, but some patients ask if they can take a particular medication. It is difficult for me to answer, so I refer them to a doctor”.

Nurse-20.

Quality indicators help assess the achievement of nursing care goals and identify areas for improvement and development in nursing practice. The absence of clear quality indicators for nursing consultations leads to misunderstanding among nurses about the outcomes of their work. Most nurses still do not fully understand how the quality of their work is evaluated, as evidenced by the following statements:

“The quality of our work is assessed based on patient appointments (registration), completion of questionnaires, and referrals to specialists. They check whether the questionnaires (related to diabetes, cancer, etc.) are filled out accurately. If we submit our work reports on time, have no patient complaints, and maintain good relationships with patients, then our work is evaluated positively. For example, if we are supposed to see more than 10 patients in one day, the quality of these appointments is evaluated”.

Nurse-21.

“…I do not know, but at least we assess quality based on patient complaints, but there have not been any complaints as such”.

Nurse-22.

## 4. Discussion

Based on the results of our study, it is evident that APNs within the primary healthcare system in Kazakhstan are still largely in the implementation phase. Our research, the first of its kind in Kazakhstan, focused on the practice of independent nursing consultations and consequently identified several issues: low awareness among the public and nurses about the role of APNs, a lack of complete trust from both patients and physicians, insufficient knowledge and skills among nurses in establishing nursing diagnoses and prescribing medications, an inadequately adapted work environment for independent nursing consultations, and the absence of clear quality indicators for nursing consultations.

Research indicates that the low level of public awareness about the role of APNs limits their potential. Patients often do not understand the range of services APNs can provide, leading to mistrust and a preference for traditional physician consultations. A review by Laurant et al. [21] demonstrated that most patients are unaware of the specific role of APNs and instead assess them based on their perceived competence. These findings closely align with our research, which we attribute to the relatively recent introduction of APNs in some clinics in the Republic of Kazakhstan. Consequently, many patients initially lacked understanding of the scope of independent nursing consultations. Others confirmed that during the initial phase of implementing a new nursing model, there is general uncertainty, which often influences patients’ choice between consulting a physician or an APN [22]. In a 2019 study, Gysin et al. [23] stated that APNs in a Swiss family practice best meet the needs of elderly, multimorbid, and complex patients. Conversely, in our study, patients preferred to consult their general practitioner in complex situations.

Additionally, another significant barrier is the insufficient knowledge and skills of nurses in making nursing diagnoses and prescribing medications. From the perspective of an experienced practicing nurse, one of the primary factors negatively impacting the implementation of the APN role was the lack of confidence in their competence or ability to fulfill their responsibilities. There was a lack of confidence in their knowledge, which affected their ability to, for example, prescribe medications or make independent decisions [24,25]. The legal role and scope of practice for APNs are not yet fully defined. Despite national efforts to establish a nursing regulatory framework, not all countries have implemented legislation granting APNs the authority to prescribe medications [26,27,28]. Research by Kroezen has shown that the United Kingdom has extensive experience with nurse prescribing, where two distinct models of nurse prescribing have been developed: independent and supplementary. In the independent model, nurses are authorized to prescribe medications on their own, including the initial prescription of a drug. The supplementary model involves nurses continuing to prescribe medications after a physician has made a diagnosis and initiated treatment [29]. In contrast, our study found that nurses pointed to the lack of legal authority to prescribe medications and attributed this to insufficient competence in pharmacology. The lack of knowledge and skills causes concern and reduces the likelihood that nurse prescribers will take responsibility for their medication prescribing decisions [30]. This issue can be addressed by ensuring that APNs have access to appropriate education, professional training, and support [31]. For instance, in Poland, a country where nurses were recently granted legal authority to prescribe medications, a lack of awareness among nurses about the role and regulations related to prescribing, as well as their poor readiness to assume this role, were among the factors and barriers to implementing nurse prescribing plans [32]. Other barriers to medication prescribing in Iran included the physician-dominated culture of the healthcare system and conflicts of interest between physicians and nurses. Physician resistance has consistently been regarded as a significant obstacle to nurse prescribing. Physicians typically occupy the top of the healthcare hierarchy and seek to protect their authority and power [33].

One of the barriers we identified in our study that hinders the effective work of APNs is an unadapted work environment. Our findings are consistent with those of Claire Torrens [34], who mentioned issues related to the physical environment (i.e., working conditions), including the lack of infrastructure to support APN positions. In the above studies, working conditions were described as particularly challenging, such as the shortage of physical space (e.g., rooms) to accommodate advanced practice nurses who could conduct patient consultations. In our research, nurses noted that the lack of private offices for independent consultations prevents them from fully addressing all patient complaints, which leads to a decrease in the accuracy of nursing diagnoses.

### Limitations

We conducted a study only in a few polyclinics where independent nursing is performed. Although we conducted interviews with a small number of nurses, it still allowed us to have an in-depth discussion and obtain meaningful answers.

## 5. Conclusions

Our analysis highlights numerous barriers to the development of the APN role. Therefore, it is crucial to provide regulatory support for the autonomy of APNs. Clear quality indicators for nursing consultations need to be developed, and the work environment should be adapted to meet the needs of independent nursing practice by providing the necessary resources. Equally important is the enhancement of nurses’ professional skills through regular training and continuing education, both in Kazakhstan and abroad. Efforts should also be made to increase public awareness of the APN role and to build trust among patients and physicians. A comprehensive approach to addressing these challenges will elevate the status of APNs and ensure the delivery of high-quality and effective healthcare services.

## Figures and Tables

**Table 1:** Questions for semi-structured interview with APNs.

No.	Questions
1	Gender of the respondent
2	Age of the respondent
3	Length of service in the position of extended practice nurse
4	Education of the respondent
5	From which sources do patients most often learn about independent nursing consultations?
6	What are the main functional duties performed by nurses during independent consultations?
7	Do you use the 5 steps of the nursing process in your practice?
8	Do you know what a “nursing diagnosis” is? Do you use the international classification of nursing diagnoses?
9	How much time does a consultation with one patient take?
10	What difficulties or challenges do you encounter during patient consultations?
11	How often do you seek assistance from a doctor during a consultation? What are the most common issues for which you seek help?
12	Do you feel adequately knowledgeable and skilled to conduct independent consultations? If not, what specific knowledge or skills do you feel are lacking?
13	Please share both the positive and negative aspects of conducting independent appointment?
14	Which programs and modules in the medical information system do you use when conducting independent consultations?
15	How do patients perceive independent nursing appointment?
16	How is the quality of your work evaluated?
17	Are you satisfied with your working conditions? If NO, please list the reasons for your dissatisfaction.
18	What suggestions do you have for improving the organization of independent nursing appointment?

**Table 2:** Demographic characteristics of participants.

Respondents	Structure	Absolute Number	%
Gender	male	2	9
	female	20	91
Age	18 to 29 years old	10	45
	30 to 39 years old	5	23
	40 to 49 years old	3	14
	50 to 63 years old	4	18
Length of service in the position of extended practice nurse	year	17	77
	From one year and above	5	23
Education	Higher education	21	90
	Postgraduate	1	10
Total:		22	100%

**Table 3:** Main themes and subthemes.

Themes	Subthemes
People awareness of the role of an independent nursing appointment	Low patient attendance at independent nursing appointments. Insufficient knowledge or misunderstanding among patients about the role of APNs. Incomplete trust of patients in APNs.
Adaptation of the work environment	A large number of medical information programs and a lack of integration between them. Lack of separate offices for independent consultations.
Confidence of nurses to provide independent services	Insufficient competence in the field of pharmacology. Insufficient competence in making a nursing diagnosis. Lack of clear indicators of the quality of independent nursing appointments.

## Data Availability

All data generated or analyzed during this study are included in this published article.
